# Anchor objects guide spatial attention during visual search

**DOI:** 10.3758/s13414-025-03198-0

**Published:** 2025-12-02

**Authors:** Makayla Souza-Wiggins, Joy J. Geng

**Affiliations:** 1 Center for Mind and Brain, University of California, 267 Cousteau Pl, Davis, CA USA; 2 Department of Psychology, University of California, Davis, CA USA

**Keywords:** Attentional guidance, Visual search, Anchor objects, Semantics

## Abstract

Semantic information is used to guide attention during scene-viewing, but how exactly does this occur? We tested the hypothesis that “anchor objects” are a special class of semantic associate essential to efficient target search in naturalistic scenes. Adult participants completed a RSVP search task for a target object preceded by a prime. In Experiment 1a (*N* = 210), the prime was either a semantic anchor or an unrelated object followed by a target in a congruent or incongruent spatial location relative to the prime. Experiment 1b (*N* = 157) served as a direct replication of Experiment 1a with a more selective stimulus set. In Experiment 2 (*N* = 158) the prime and target objects were reversed to assess the directionality of the effects. Results showed that semantically related objects facilitated target identification no matter which object was the prime. However, only anchor objects acted as predictive spatial cues, relative to control object pairs. The results indicate that anchor objects are a unique type of semantic associate, providing information about “what” objects are nearby and “where” they might be. The role of anchor objects in predicting the location of associated objects goes beyond both general scene regularities (e.g., “small things go on top of big things”) and semantic associations (e.g., pots and stoves).

**Supplementary Information:**

The online version contains supplementary material available at 10.3758/s13414-025-03198-0.

## Introduction

Imagine stepping into an unfamiliar kitchen, tasked with finding wineglasses. You might instinctively look toward surfaces or storage cabinets, using scene structure and knowledge of the target object features to guide your attention (Biederman, 1972; Boettcher et al., 2018; Brockmole et al., 2006; Castelhano & Krzyś, 2020; Hayes & Henderson, 2019; Henderson et al., 1999; Peacock et al., 2019). You might also use objects related to the wineglasses, such as a wine cabinet, as proxies for the smaller, difficult-to-find wineglasses. These kinds of object-to-object associations may provide meaningful cues that can guide attention toward likely target locations. But how, exactly, do these semantic relationships between objects affect search? We address this question by investigating how associations between thematically related objects—those with high co-occurrence statistics within natural contexts—aid target search. Previous research has shown that thematically related objects prime each other’s identity (Green & Hummel, 2006; Krugliak et al., 2024; Mack & Eckstein, 2011; Mirman et al., 2017; Nah & Geng, 2022; Shomstein et al., 2019; Torralba et al., 2006; Turini & Võ, 2022). Here we test the hypothesis that the pairwise object benefits go beyond identity priming and include proactive spatial predictions of the target’s relative location.

There is a subtype of objects within scenes, known as “anchor” objects, that are particularly important for creating scene structure and spatial organization (Boettcher et al., 2018; Draschkow & Võ, 2017). Anchors are large, static objects (e.g., a stove) that provide information about the spatial layout of smaller related objects (e.g., a pot; Helbing et al., 2022; Võ et al., 2019). The information provided by anchor objects contrasts with that of smaller semantic associates (e.g., pan and pot), which, despite having high co-occurrence and semantic relatedness, do not provide strong information about each other’s spatial positioning—perhaps due to the high degree of variability in their own locations. Evidence that anchor objects facilitate search comes from studies showing that less of a scene needs to be searched through when an anchor object is present (Boettcher et al., 2018; Hebling et al., 2022). For example, Hebling et al. (2022) instructed participants to find target objects, such as a pretzel, in a virtual kitchen. Anchor objects were placed in consistent locations with a typical kitchen layout (e.g., the refrigerator on the ground along counters) or inconsistent locations (e.g., the refrigerator on the counter). The results showed that across a number of eye, head, and body metrics, search and localization of the target was always more efficient when the scene anchors and target objects were in their naturally occurring places. These findings suggest that anchor objects influence the search process by providing a spatial syntax for the room that organizes search for other smaller objects (Võ & Wolfe, 2013).

However, in addition to anchor objects facilitating search by providing a spatial structure to rooms, they may also carry one-to-one relational spatial predictions for where semantically associated objects should be located. If the latter is true, then we would expect anchor objects to capture attention during search for a semantically related target because they act as more easily identifiable reference points that generate a spatial cue for where the target is likely to be. In Experiments 1a and 1b, we test this hypothesis by manipulating the spatial congruency between an anchor prime and a target object in a rapid serial visual presentation (RSVP) search task devoid of other scene information. In Experiment 2, we reversed the roles of anchor primes and targets to assess whether smaller “non-anchor” objects, previously serving as targets, also act as spatial predictors for anchor objects.

These experiments reveal that anchor objects play a special role as a relational spatial cue for the target’s location, over and above its role in priming object identity and providing scene structure more generally. Such a result goes beyond what we already know by showing that anchor objects facilitate search by providing direct object-to-object spatial predictions about relative location. Understanding the interaction between semantic relatedness and spatial predictability offers deeper insights into the mechanisms that guide visual attention, helping to explain how we efficiently navigate complex environments where targets must be identified amidst a backdrop of visual clutter and complexity.

## Experiment 1

Experiments 1a and 1b used a 2 × 2 design to test whether anchor objects prime target identity and predict the target’s location. Experiment 1a is the original study and Experiment 1b is a replication. We hypothesize that targets appearing in a relative location predicted by the “anchor” prime object will be detected faster and more accurately than targets appearing in an equally close and physically plausible, but spatially incongruent, location. Furthermore, we expect that immediate memory of where the target was located will be influenced by the anchor’s location, reflecting how expectations prime search and also bias subsequent memory. The results will clarify how an anchor object’s ability to predict a smaller target object’s location contributes to efficient visual search.

### Method

#### Participants

Undergraduate students from the University of California, Davis, were recruited through the UC Davis SONA system to participate. Data were collected online using the Testable platform (Testable.org). A total of 210 participated in Experiment 1a (104 in Version 1 and 106 in Version 2) after exclusion criteria were applied. Twenty-one participants were excluded due to accuracy below 80% on standard search trials, or for rating their effort as a 4 or below on a 6-point self-report survey administered after the experiment. The self-report effort scale was included to assess for low task-engagement since participants were recruited from the standard UC Davis subject pool and they were guaranteed course credit for participating irrespective of performance. The sample size was determined based on similar antecedent experiments (see Supplemental Materials). Of the participants, 165 participants identified as female, 40 identified as male, five identified as non-binary, with an average age of 19.7 years (see supplemental for full demographic details). All participants had normal or corrected-to-normal vision and were naive to the purpose of the experiment. Each participant provided informed consent in accordance with the guidelines set by the University of California, Davis Institutional Review Board (IRB).

The replication sample size in Experiment 1b was determined by a power analysis run in the *mixedpower* package (Kumle et al., 2021) using data from Experiment 1a. To achieve the desired power of 0.80 at a significance level of 0.05 the minimum required sample size to detect an interaction effect in this replication experiment was 155 after exclusion criteria were applied. If more were collected during a single online data acquisition period, we did not throw out additional participants. Experiment 1b had a total of 157 participants (80 in Version 1 and 77 in Version 2). Thirty-two participants were excluded after the same participant recruitment and exclusion criteria were applied as in Experiment 1a. Of the parctipants, 118 participants identified as female, 38 identified as male, one identified as nonbinary, with an average age of 19.9 years.

A separate group of participants (*n* = 80) engaged in a two-part judgment task in which we evaluated the thematic relationship and normative spatial configurations of our object pairs. This task was also conducted using the Testable platform and the same requirements for participation in the main experiment were applied.

### Stimuli and apparatus

**Fig. 1:**
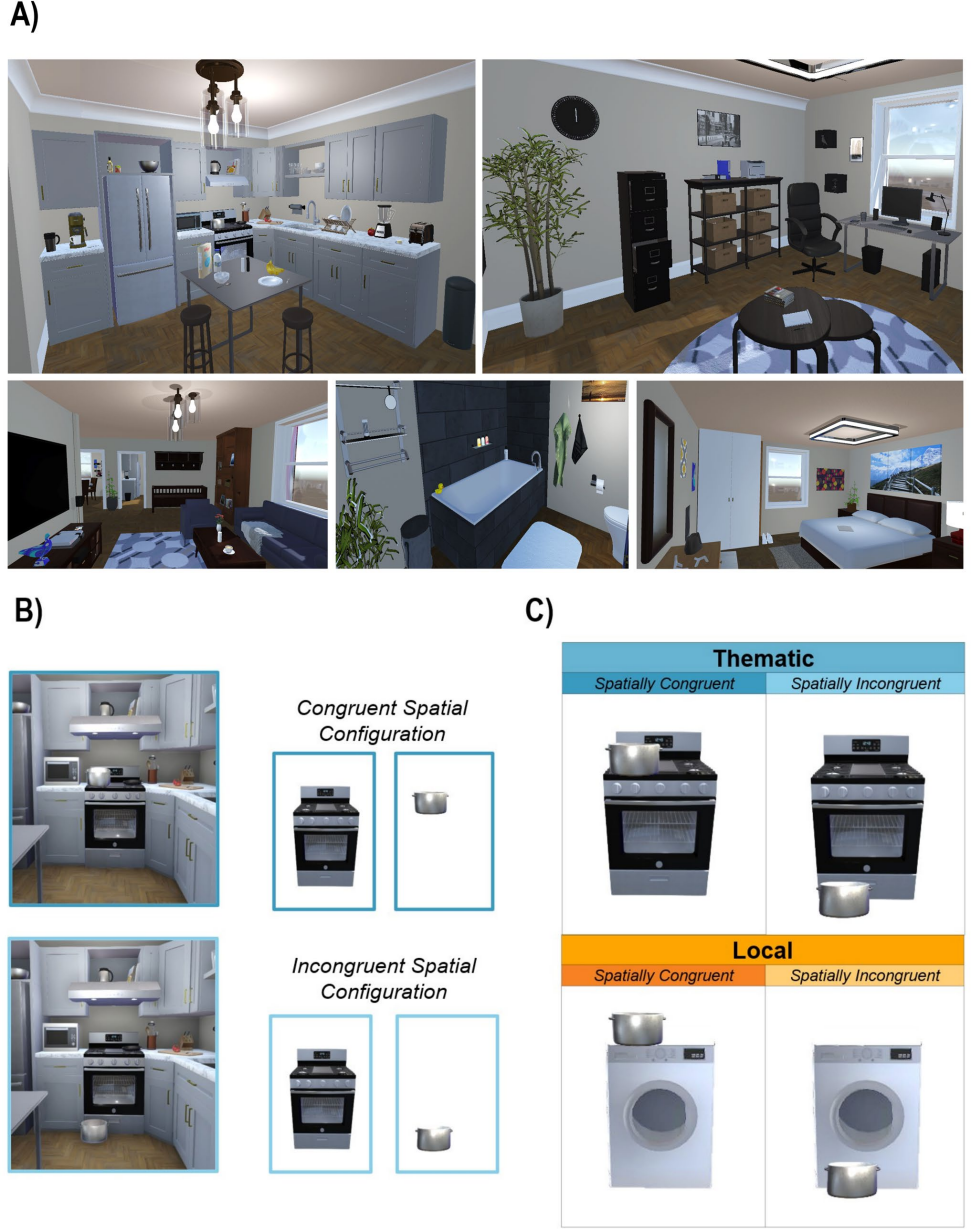
Stimuli and experimental conditions. (***a***) The five VR rooms developed in Unity that contained all the anchor objects and targets. (***b***) Illustration of how anchor and target objects were extracted from the original 3D room in Unity. After placing the objects and camera in Unity, the entire scene was turned off, with one object turned on at a time. An image was taken from the same camera position for each object. (***c***) The experimental conditions (2 X 2), showing how the objects would appear if they were presented simultaneously on screen

To maintain accurate spatial configurations and object proportions, we designed a house in virtual reality (VR) using Unity (Fig. 1a). Within this house, we placed 15 anchor objects in their designated rooms, each paired with two smaller, thematically related objects positioned either in congruent or incongruent locations relative to the anchor. For example, a stove would be placed in its designated spot in the kitchen, with a pot and pan either on the burners (congruent) or on the floor in front of the stove (incongruent). Note that incongruent pairs still obey physics and are therefore plausible in natural settings. These pairings were based on previously established anchor pairs described by Turini and Vo (2022) and were composed of an “anchor” object with a smaller “target” object. For our “local” control condition, we used the same target objects but placed them in either physically congruent or incongruent spatial configurations with an unrelated, larger, static object (e.g., the pot and pan were placed on top of, or on the ground in front of, a washing machine; see Fig. 1bc). This resulted in 15 “local anchor” objects, matching the number of ‘thematic anchor’ objects in our main condition. We then created two versions of the task to control for object effects, with each participant completing only one version. For example, in Version 1, participants saw the pot paired with the stove in the *thematic* condition and the pan paired with the washing machine in the *local* condition. In Version 2, these pairings were reversed (e.g., the pot was now in the local condition with the washing machine, and the pan was with the stove).

We then made the scene invisible in Unity, except for the anchor object, and took a picture, with the camera placed in the same location within each scene. We did the same for the target object after making the anchor object invisible using the same camera location (Fig. 1b). This method ensured that each image was captured with the objects in their correct spatial relationship relative to each other and the viewer. All stimuli were presented with a gray background.

A judgment task of all the stimuli in Experiment 1a was used to assess the naturalness of our stimulus pairings. The objects were presented for the same duration that participants saw them in the main experiment (i.e., 450 ms). The task was divided into two blocks: Block 1 was the thematic judgment task, and Block 2 was the spatial configuration task. For the thematic judgement task, participates were shown pairs of objects and asked to rate the likelihood of the two objects appearing together in natural scenes (1 = extremely unlikely, 6 = extremely likely). For the spatial judgment block, participants evaluated object pairs in different configurations and rated the likelihood of each configuration occurring in natural environments on the same 6-point scale. These ratings were used to classify the local object configurations.

Only a subset of the VR object pairings from Experiment 1a were kept for Experiment 1b based on results from the spatial and thematic judgment tasks run as part of Experiment 1a. Thematic object pairings were only included if they scored greater than or equal to a median of 4 on the 6-point scale. Conversely, local object pairings had to score at or below a median of 3 to be included. This left us with seven thematic pairings and seven local pairings in Experiment 1b.

#### Design and procedure

**Fig. 2:**
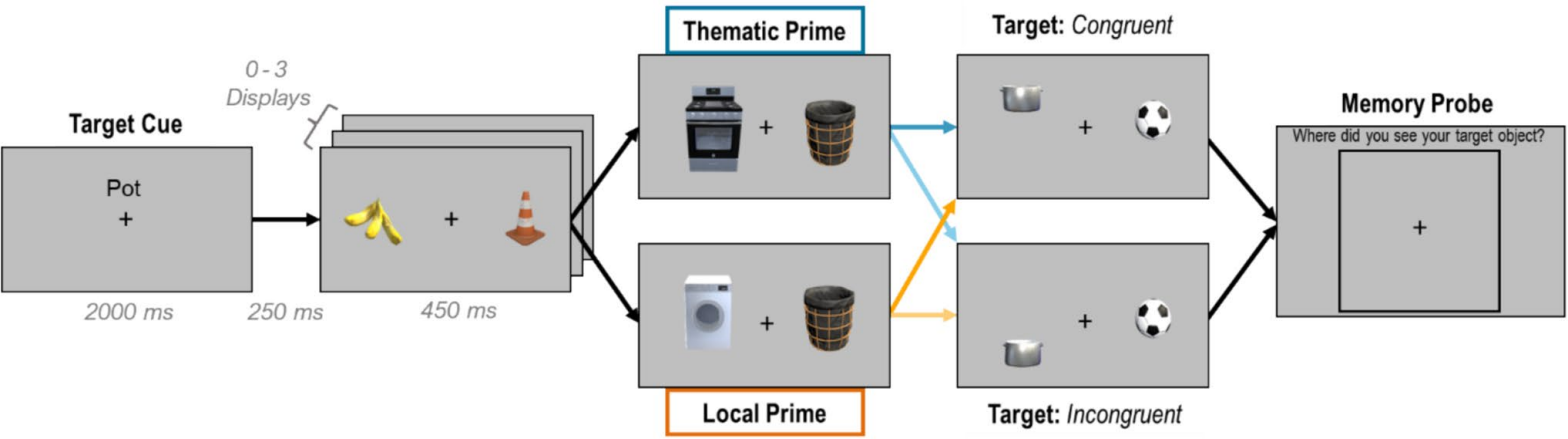
Trial Sequence for Experiments 1a and 1b. First a target cue (e.g., a pot) appeared, followed by 0–3 “filler” displays. Next, a prime object display appeared with a thematic prime (blue) or a local prime (orange). This was followed immediately by a target display with the target in a spatially congruent or spatially incongruent location relative to the prime. All displays were visible for 450 ms with a 250 ms ISI. The 4 colored arrows indicate the possible transitions between prime and target displays and illustrate the 2 × 2 experimental design. Participants responded by pressing a button to indicate the target’s location (left or right). After participants responded to the target’s location, a memory probe display appeared, asking participants to indicate with a mouse click where the target had appeared on the screen. Note: Object stimuli were jittered in a circular pattern to reduce predictability, and object size is enlarged in this figure for visualization purposes. (Color figure online)

The design and procedures were identical for Experiments 1a and 1b. We manipulated the semantic relatedness and spatial congruency of object primes in a RSVP task in a 2 (prime object: *thematic* or *local*) × 2 (spatial congruency: *congruen*t or *incongruent*) design. The reliability of the RSVP task paradigm as a semantic priming task was validated in a separate experiment that confirmed that thematic primes capture attention (see Supplemental Materials). At the start of each trial in this experiment, a target cue word was presented for 2,000 ms (e.g., “Pot”). Following this, between 0 to 3 “filler” displays with two lateralized objects were shown in sequence (Fig. 2). This variable number of filler displays was used to ensure that the onset of the prime object and target displays remained unpredictable. After a randomly determined number of filler displays, a display with the prime object appeared followed by the target display. The location of the prime object on the left or right was randomized and the specific location was jittered, but the target was always on the same side as the prime. All displays lasted 450 ms (or terminated when a response was made) and were separated by a 250-ms interdisplay interval. The prime object could either be a *thematic prime* (e.g., “stove”) or an unrelated *local prime* (e.g., “washing machine”). Importantly, the target object (e.g., “pot”) appeared in either a *spatially congruent* or *spatially incongruent* location relative to the prime object. The order in which trials from all conditions were presented was randomized. Each of the 30 targets in Experiment 1a and the 14 targets in Experiment 1b was presented six times (three times in its *congruent* configuration and three times in its *incongruent* configuration), totaling 180 trials per participant in Experiment 1a and 84 in Experiment 1b. Filler display objects and the prime stimuli were randomly jittered in a circular pattern around the fixation point but the placement of the target relative to the prime was always fixed based on their natural positions in the Unity scene.

Participants were asked to report whether the target appeared on the left or right side of the fixation using the left and right arrow keys on their keyboard. To be sure that their responses were based on actual detection of the target, and not a guess based on the location of the prime object, we added a spatial memory probe to the end of each trial. Participants were presented with the outline of a large square and instructed to click inside the square where the target object appeared. We expected that memory would be more precise for targets that were better encoded. Participants completed four practice trials before starting the main task. Upon completion, participants filled out a short survey. They were asked the following questions: “how much effort did you put into completing this task on a scale of 1 to 7”; “did you use any strategies to find the target objects? If so, please explain your strategy”; and “during the task, certain objects appeared right before your “target” object. Did you notice any of these preceding objects? If yes, please try to list as many as you can remember along with the following target.”

### Data analysis

Data from Experiments 1a and 1b were analyzed using a custom script in R Studio. The experiment followed a 2 × 2 design, with the prime condition (*thematic* or *local*) and spatial congruency (*congruent* or *incongruent*) as the two independent variables. RT data were analyzed using a linear mixed-effects regression model using the “lmer” function (Bates et al., 2015). The model included prime object and spatial congruency as fixed effects. To account for baseline differences between individuals, participants were modeled with random intercepts. Incorrect trials and trials with RTs less than 200 ms were removed from the RT analysis to account for anticipatory responses. Additionally, an exploratory linear mixed-effects regression was run to analyze the impact of repeated target occurrences on reaction time, motivated by prior evidence of learning effects in similar object-priming tasks (Gronau et al., 2008). This model included target occurrence (i.e., the number of times that a specific prime–target pair was shown over the course of the experiment), prime type, and spatial congruency as fixed effects, and random intercepts were included for participants. To examine learning over time, this analysis divided the data up into six bins based on target occurrence. This resulted in 15 total trials for each target occurrence level in Experiment 1a and seven trials per level in Experiment 1b. There were 210 participants in Experiment 1a and 157 in Experiment 1b. Effect sizes for linear mixed effects regressions were not reported due to the complexity of interpreting these measures in the context of the model (Meteyard & Davies, 2020).

The accuracy data was analyzed using a logistic mixed-effect logistic regression model with the same fixed effects and random intercepts as the RT analysis. Lastly, to analyze the memory probe data, we used a linear mixed-effects model to examine how the prime condition and spatial congruency influenced click accuracy. Fixed effects included the prime and spatial conditions, while random intercepts were included for participants and target objects. The target object was included to account for variability in the strength of spatial predictiveness across different object pairings. The primary outcome measure was the average Euclidean distance between the target object’s location on the screen and the participants’ mouse click as a measurement of the accuracy of their recall of the target’s location.

## Results

### Thematic primes generate stronger spatial expectations for target location

**Fig. 3:**
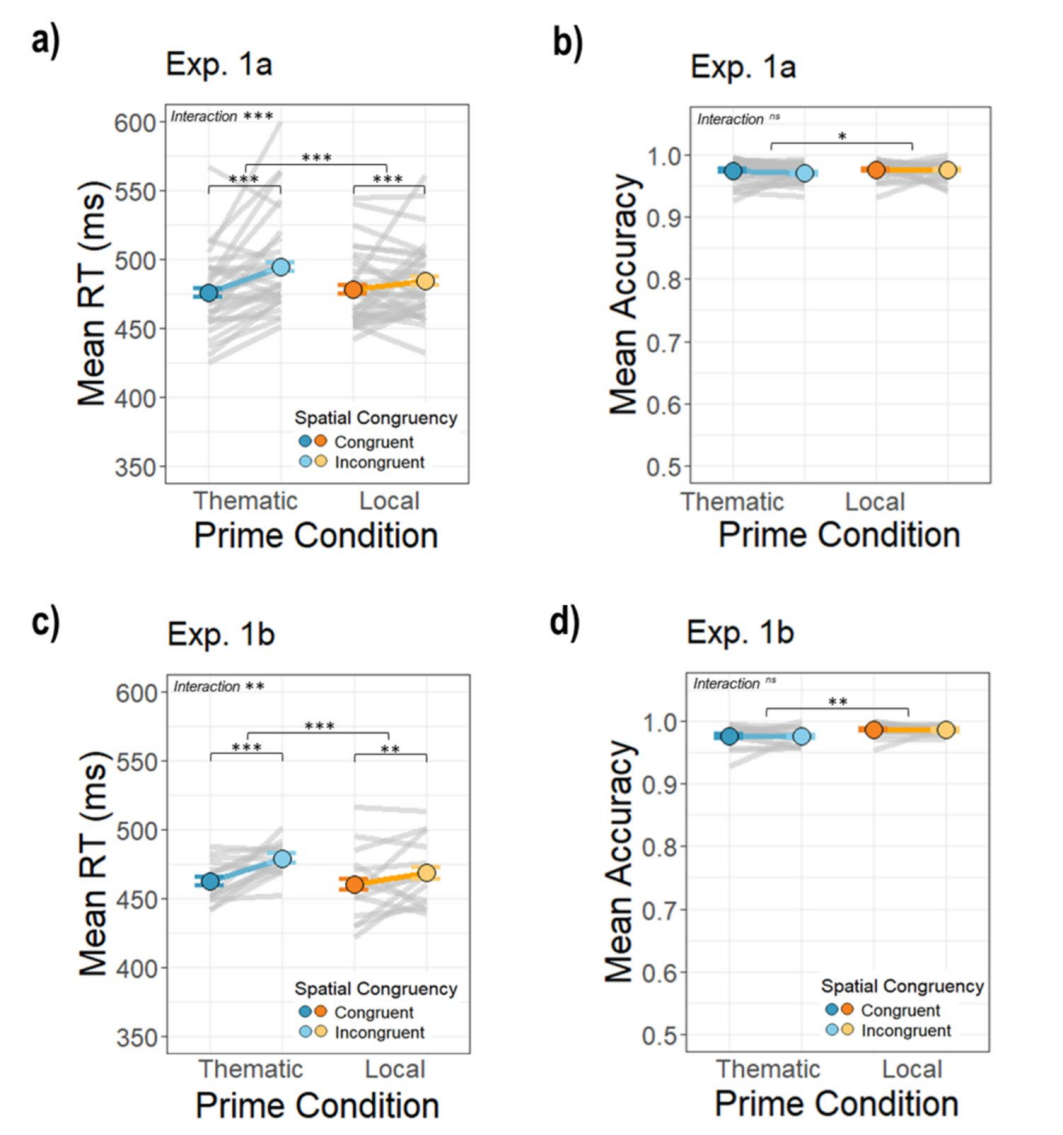
Average reaction times and accuracy in each condition for Experiments 1a and 1b. Colored dots represent mean values for each condition and the gray lines represent averages across participants for each prime-target pairing. The error bars represent the standard error of the mean across participants in each condition. Data are plotted on the same scale as for Experiment 1a and Experiment1b to facilitate comparison. (Color figure online)

The RT results confirmed our hypothesis that anchor objects prime smaller semantically related objects, with the benefits of thematic primes being strongest when spatial configurations are naturalistic. Consistent with this, there was a significant interaction between the prime condition and spatial congruency in Experiment 1a (β = −12.86, *SE* = 2.22), *t*(33390.69) = −5.79, *p* <.001, and Experiment 1b (β = −8.16, *SE* = 3.5), *t*(12013.01) = −2.35, *p* =.019. The interactions were due to a larger difference between the spatially *incongruent* and *congruent* targets in the *thematic* condition compared to the *local* condition; anchor primes produced stronger spatial expectations for the relative location of the target (Experiment 1a *thematic congruent: M* = 475.75, *SD* = 43.68*, thematic incongruent: M* = 494.67, *SD* = 45.14, *local congruent*: *M* = 477.33, *SD* = 42.91, *local incongruent:* 484.19, *SD* = 46.80; Experiment 1b *thematic congruent: M* = 462.60, *SD* = 40.84, *thematic incongruent: M* = 479.22, *SD* = 45.03, *local congruent*: *M* = 460.00, *SD* = 49.39, *local incongruent: M* = 468.43, *SD* = 50.95; see Supplemental Materials for all pairwise comparisons; Fig. 3ac). These results are consistent with our hypothesis that thematic primes facilitate target detection because they serve as spatial cues for where the target should be.

The main effects of prime condition and spatial congruency were also significant. Participants responded slower overall in the *thematic prime* condition compared with the *local prime* condition in both experiments, Experiment 1a: β = 11.12, *SE* = 1.57), *t*(33390.73) = 7.07, *p* <.001; Experiment 1b: β = 10.92, *SE* = 2.46, *t*(12013.00) = 4.434, *p* <.001. This effect was largely driven by long RTs in the spatially incongruent *thematic prime* condition. The significant main effect of spatial congruency on RT showed that participants responded faster in the *congruent* compared to the *incongruent* spatial condition in both experiments, Experiment 1a: β = −6.38, *SE* = 1.56, *t*(33390.78) = −4.09, *p* <.001; Experiment 1b: β = −8.28, *SE* = 2.45, *t*(12013.2) = −3.38, *p* <.001, suggesting a general preference for natural configurations with larger objects beneath smaller objects.

### Performance accuracy is near ceiling in all conditions

Accuracy was high across all conditions (*thematic congruent:* Experiment 1a: *M* = 0.98, *SD* = 0.03; Experiment 1b: *M* = 0.98, *SD* = 0.04, *thematic incongruent:* Experiment 1a: *M* = 0.97, *SD* = 0.03; Experiment 1b: *M* = 0.98, *SD* = 0.04, *local congruent:* Experiment 1a: *M* = 0.98, *SD* = 0.03; Experiment 1b: *M* = 0.99, *SD* = 0.03, *local incongruent:* Experiment 1a: *M* = 0.98, *SD* = 0.03; Experiment 1b: *M* = 0.99, *SD* = 0.03; Fig. 3bd). The interaction between the prime and spatial congruency conditions was not significant (β = 0.17, *SE* = 0.13, *z* = 1.26, *p* =.208; β = −0.01, *SE* = 0.28, *z* = −0.02, *p* =.982). There was, however, a significant main effect of prime condition such that the *local* prime condition was more accurate than the *thematic* condition in both Experiment 1a and 1b (Experiment 1a: β = −0.21, *SE* = 0.09, *z* = −2.23, *p* =.026; Experiment 1b: β = −0.58, *SE* = 0.19, *z* = −3.00, *p* =.003). The was no main effect of spatial congruency in either experiment (Experiment 1a: β = 0.03, *SE* = 0.10,* z* = 0.29, *p* =.195; Experiment 1b: β = 0.05, *SE* = 0.22, *z* = 0.24, *p* =.808).

### Prime–target pairings are learned over time

**Fig. 4:**
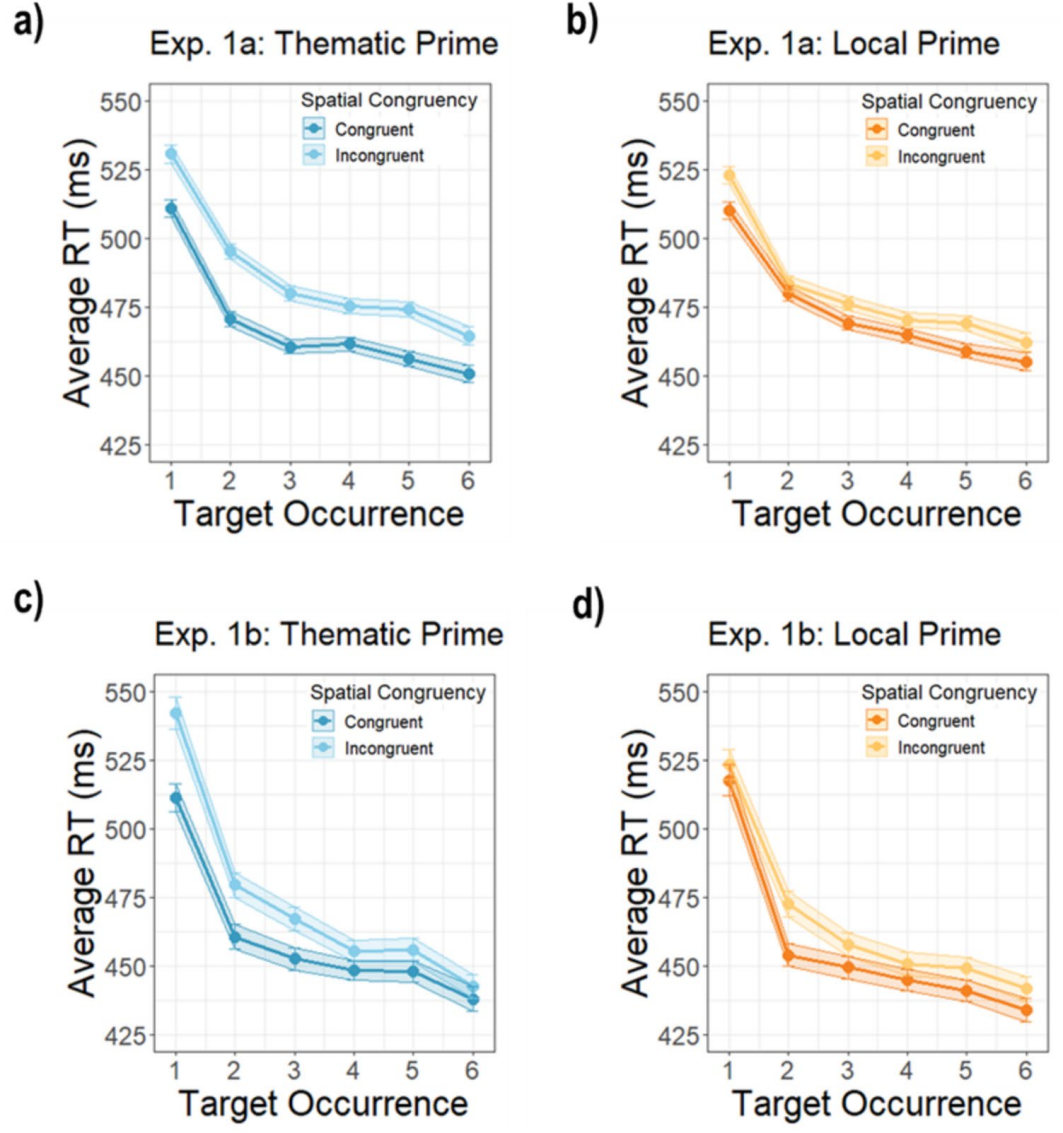
Average reaction times across target occurrences for Experiments 1a and 1b. **a)** The average RTs for thematic prime trials across target occurrences in Experiment 1a. **b)** The local prime trials. **c)** Thematic prime trials in Experiment 1b. t **d)** The local prime trials. The shading around lines indicates standard error of the mean. Data are plotted on the same scale as for Experiment 1a and Experiment 1b to facilitate comparison. (Color figure online)

To better understand how experience with prime–target pairs might have changed performance over time, we conducted an exploratory analysis of RT as a function of the prime condition × spatial congruency × target occurrence, where “target occurrence” is the number of times that a specific prime–target pair was shown over the course of the experiment. The three-way interaction between prime condition, spatial congruency, and target occurrence was not significant in Experiment 1a (β = 0.42, *SE* = 1.35), *t*(33402.06) = 0.31, *p* =.757, but was in Experiment 1b (β = 4.67, *SE* = 2.08), *t*(12033.47) = 2.25, *p* =.025 (Fig. 4). The interaction in Experiment 1b showed that while the effect of spatial congruency increased after the first prime–target occurrence in the *local* condition, it decreased in the *thematic* condition. This suggests that participants learned about the specific prime–target pairs and their possible spatial configurations as they repeated, diminishing the strong natural bias for targets in the thematically congruent configuration, and increasing expectations for the positioning of primes and targets in local pairs. The fact that this learning effect was more pronounced in Experiment 1b may be due to fewer prime–target pairs in that experiment, allowing participants to become more aware of the specific stimulus pairs.

No other effects involving target occurrence were significant. The interaction between the prime condition and target occurrence was not significant in either experiment, Experiment 1a: β = −1.03, *SE* = 0.96), *t*(33396.04) = −1.08, *p* =.282; Experiment 1b: β = −2.73, *SE* = 1.46, *t*(12033.64) = −1.87, *p* =.062, nor was the interaction between the spatial congruence condition and target occurrence, Experiment 1a: β = 0.12, *SE* = 0.95, *t*(333402.64) = 0.13, *p* =.899; Experiment 1b: β = −0.58, *SE* = 1.46, *t*(12033.10) = −0.4, *p* =.67. However, as expected based on the previous RT analysis, the two way interaction between prime condition and spatial congruency was again significant in both experiments, Experiment 1a: β = −13.88, *SE* = 4.90, *t*(33399.44) = −2.83, *p* =.005; Experiment 1b: β = −22.80, *SE* = 7.67, *t*(12029.47) = −2.97, *p* =.025, due to a larger congruency effect in the *thematic* condition.

### Memory for the target location is more accurate with a thematic anchor prime

**Fig. 5:**
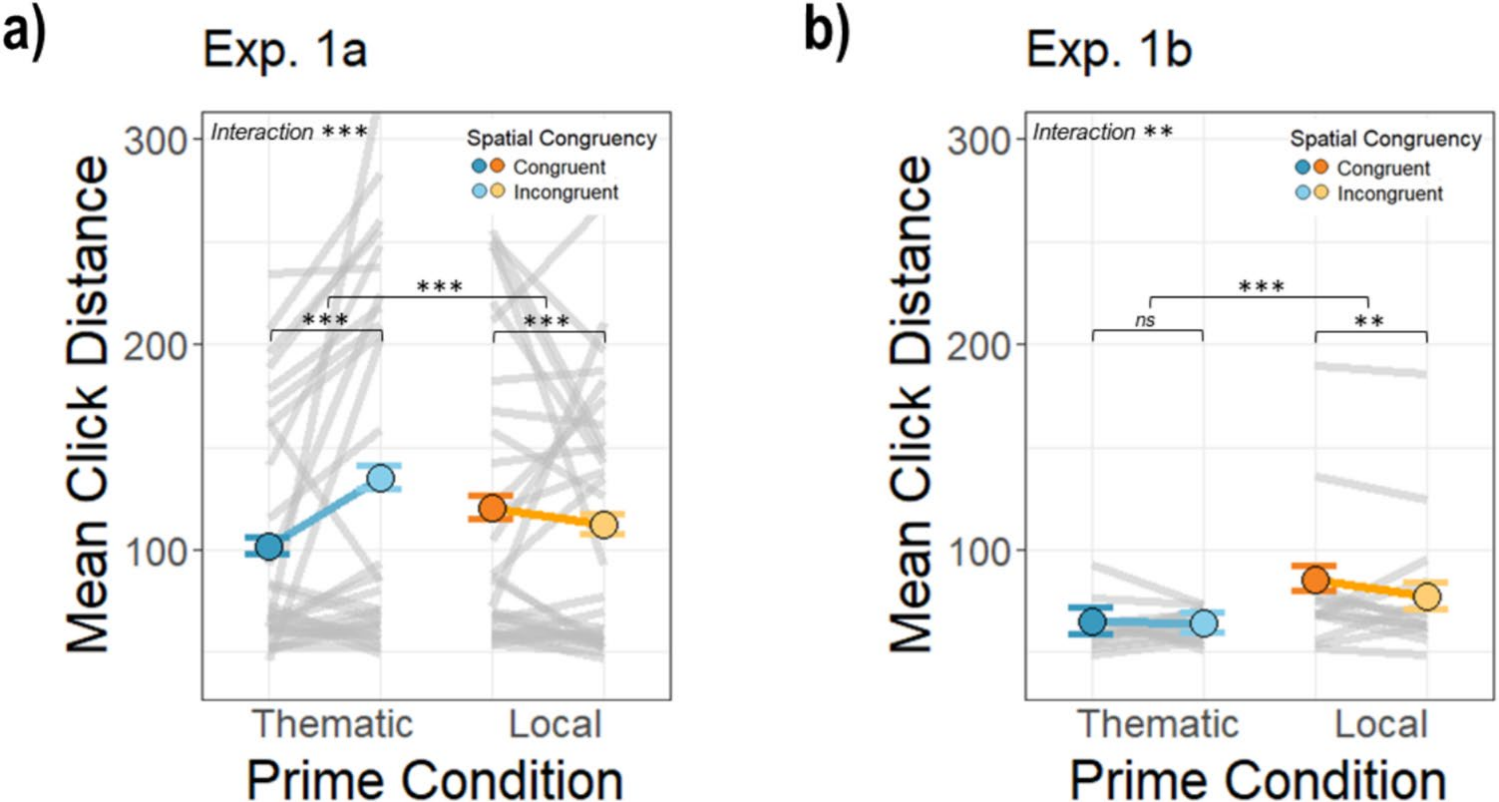
Average click distance from the actual target location across conditions for Experiments 1a and 1b. The dots represent the overall mean distance between click responses and the targets’ true location on the screen and the gray lines represent average distances for each target–anchor pairing. The error bars represent the standard error of the mean across participants for each condition. (Color figure online)

The memory probe occurred after the target display and asked participants to reproduce the location of the target. The click distance from the true target location was taken as an indicator of how accurately the target was remembered. The results showed that, similar to the RT data, there was a significant interaction between the prime condition and spatial congruency in both experiments, Experiment 1a: β = −40.59, *SE* = 1.20,* t*(33361.45) = −33.72, *p* <.001; Experiment 1b: β = −7.48, *SE* = 2.48,* t*(4280.68) = −3.01, *p* =.003. The interaction in Experiment 1a was due to a congruency effect being present in the *thematic* condition and a reversed congruency effect in the *local* condition (*thematic congruent: M* = 100.21, *SD* = 62.85; *thematic incongruent: M* = 132.55, *SD* = 87.36, *local congruent*: *M* = 117.88, *SD* = 83.95, *local incongruent: M* = 110.59, *SD* = 69.66; Fig. 5a). The interaction in Experiment 1b was due to the lack of a congruency effect in the *thematic* condition and a reversed congruency effect in the *local* condition (*thematic congruent: M* = 65.03, *SD* = 80.78, *thematic incongruent: M* = 64.01, *SD* = 61.13, *local incongruent*: *M* = 77.22, *SD* = 79.20, *local congruent: M* = 85.59, *SD* = 79.05; Fig. 5b).

There was a main effect of prime condition in both experiments. Click distances were overall smaller (i.e., accuracy was higher) in the *local* prime condition for Experiment 1a (β = 22.58, *SE* = 0.85), *t*(33361.36) = 26.47, *p* <.001, but the reverse was true in Experiment 1b (β = −12.02, *SE* = 1.89), *t*(4290.11) = −6.37, *p* <.001. There was also a significant main effect of spatial congruency in both Experiment 1a (β = 7.22, *SE* = 0.85), *t*(3361.20) = 8.53, *p* <.001, and Experiment 1b (β = 5.58, *SE* = 1.77), *t*(4281.11) = 3.16, *p* =.002. Overall, the data from the memory probes suggest that the thematically related prime anchored memory of the target location, but the exact effect of congruency on memory in the thematic condition was not consistent across experiments, perhaps due to a ceiling effect on performance in 1b. In contrast, the location of incongruent targets was consistently remembered better in the local condition.

## Experiment 2

The results from Experiments 1 were consistent with the notion that thematically related anchors act as both semantic and spatial primes for detecting smaller target objects. However, the use of only “anchor objects” as primes leaves open the question of whether the observed effects are driven by the characteristics of the anchor objects themselves—large, static, reliable objects—or merely by the semantic and spatial relatedness of any two thematic pairs. To address this, we reversed the roles of the anchors and targets from Experiment 1b so that the previous targets were now “primes” and the previous anchor primes were now the targets. If anchor objects are unique in the spatial predictions they generate, then we expect that reversing their order will reduce the effects in the thematic condition only, suggesting an asymmetry in spatial prediction. However, if the effects are the same, it would imply that all objects generate relational spatial predictions for co-occurring objects.

### Method

#### Participants

Based on the power analysis conducted for Experiment 1b, our goal was to reach a minimum of 155 participants. The same participant recruitment and exclusion criteria were applied as previously. Thirty-four participants were excluded from the analysis due to poor performance and low effort self-ratings. The final sample included 158 people (Version 1: 79; Version 2: 79), 122 identified as female, 30 identified as male, six identified as nonbinary. The average age was 19.8 years. Each participant provided informed consent in accordance with the guidelines set by the University of California, Davis, Institutional Review Board (IRB).

### Stimuli and apparatus

We stimuli were the same as those in Experiment 1b.

### Design and procedure

**Fig. 6:**
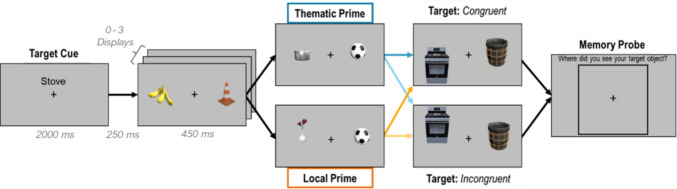
Trial Sequence for Experiment 2. First a target cue (e.g., a pot) appeared, followed by 0–3 filler display displays (each visible for 450 ms followed by a 250-ms ISI). Next, a prime appeared, which was either thematically related to (blue) or locally associated with (orange) the target. This was followed by the presentation of a spatially congruent or spatially incongruent anchor object target (the four colored arrows indicate the 2 × 2 experimental design). Participants responded by pressing a button to indicate the target’s location (left or right). Lastly, they responded to a memory probe to indicate where the target appeared on the screen. Note: Object stimuli were jittered in a circular pattern to reduce predictability, and object size is enlarged in this Fig. for visualization purposes. (Color figure online)

The control study had the same design as Experiment 1ab, maintaining a 2 × 2 within-subjects repeated-measures design with factors prime (*thematic, local*) and spatial congruency (*congruent, incongruent*). However, the roles of anchors and targets were reversed. Specifically, the targets in Experiment 1b now served as the prime stimuli, and the anchor stimuli were now the targets. For example, the “pot” was now the prime and preceded a thematically or locally related target, such as a stove or washing machine, respectively (Fig. 6).

### Data analysis

The same analyses were conducted as those in Experiment 1.

### Results

#### Spatial congruency no longer differs between local and thematic primes

In contrast to Experiments 1ab, the interaction between the prime and spatial conditions was no longer statistically significant (β = 6.03, *SE* = 3.65), *t*(12026.544) = 1.65, *p* =.098 *(thematic congruent: M* = 454.51, *SD* = 29.25, *thematic incongruent: M* = 465.66, *SD* = 30.62, *local congruent: M* = 460.15, *SD* = 24.32, *local incongruent*: *M* = 478.36, *SD* = 27.56). Unlike Experiment 1, there was no statistical difference in the spatial priming effect between *thematic* and *local* object pairs when the prime was the small object and the target was the anchor object. Shorter RTs were found for the congruent configuration for both thematic and local pairs. A direct comparison of the RT results from Experiment 1b and 2 using a mixed-effects analysis revealed a significant three-way interaction between prime condition, spatial congruency, and experiment (β = 14.19, *SE* = 5.04),* t*(24039.49) = 2.82, *p* =.005, confirming the difference between experiments in the significance of the prime × spatial congruency interaction.

**Fig. 7:**
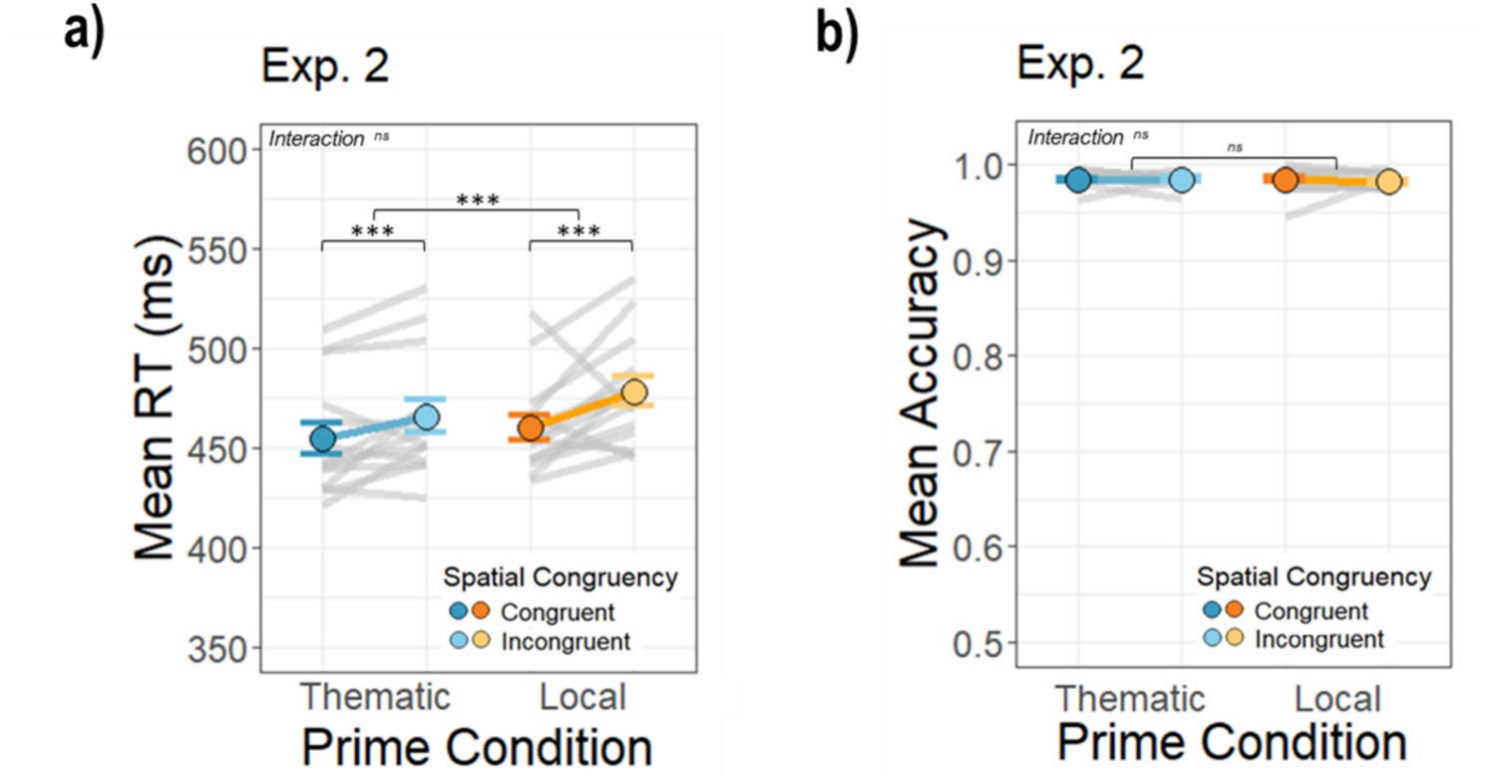
Average reaction times and accuracy across conditions for Experiment 2. Colored dots represent mean values for each condition and the gray lines represent averages across participants for each prime–target pairing. The error bars represent the standard error of the mean across participants in each condition. Data are plotted on the same scale as Experiment 1 to facilitate comparison. (Color figure online)

Similar to Experiment 1, however, there was a significant main effect of prime condition (β = −11.62, *SE* = 2.59), *t*(12027.068) = −4.49, *p* <.001. Participants responded faster in the *thematic* condition compared to *local* one, consistent with an overall facilitation based on semantic relatedness. There was also a significant main effect of spatial congruency (β = −17.57, *SE* = 2.60), *t*(12026.856) = −6.76, *p* <.001. RTs were shorter for *congruent* spatial configurations compared to *incongruent* ones for both thematic and local conditions, potentially due to the physical plausibility of little things tending to be on top of bigger things (Fig. 7a).

#### Accuracy is near ceiling in all conditions

As before, participants performed with high accuracy in all conditions (*M* in each condition was the same: 0.98, *SD* = 0.03; Fig. 7b). There were no significant effects (main effect of prime condition: β = 0.15, *SE* = 0.20, *z* = 0.76, *p* =.447, spatial congruency: β = 0.12, *SE* = 0.20, *z* = 0.59, *p* =.557, two-way interaction: β = −0.11, *SE* = 0.29, *z* = −0.39, *p* =.694).

#### Local pairs of prime–target occurrences are learned over time

**Fig. 8:**
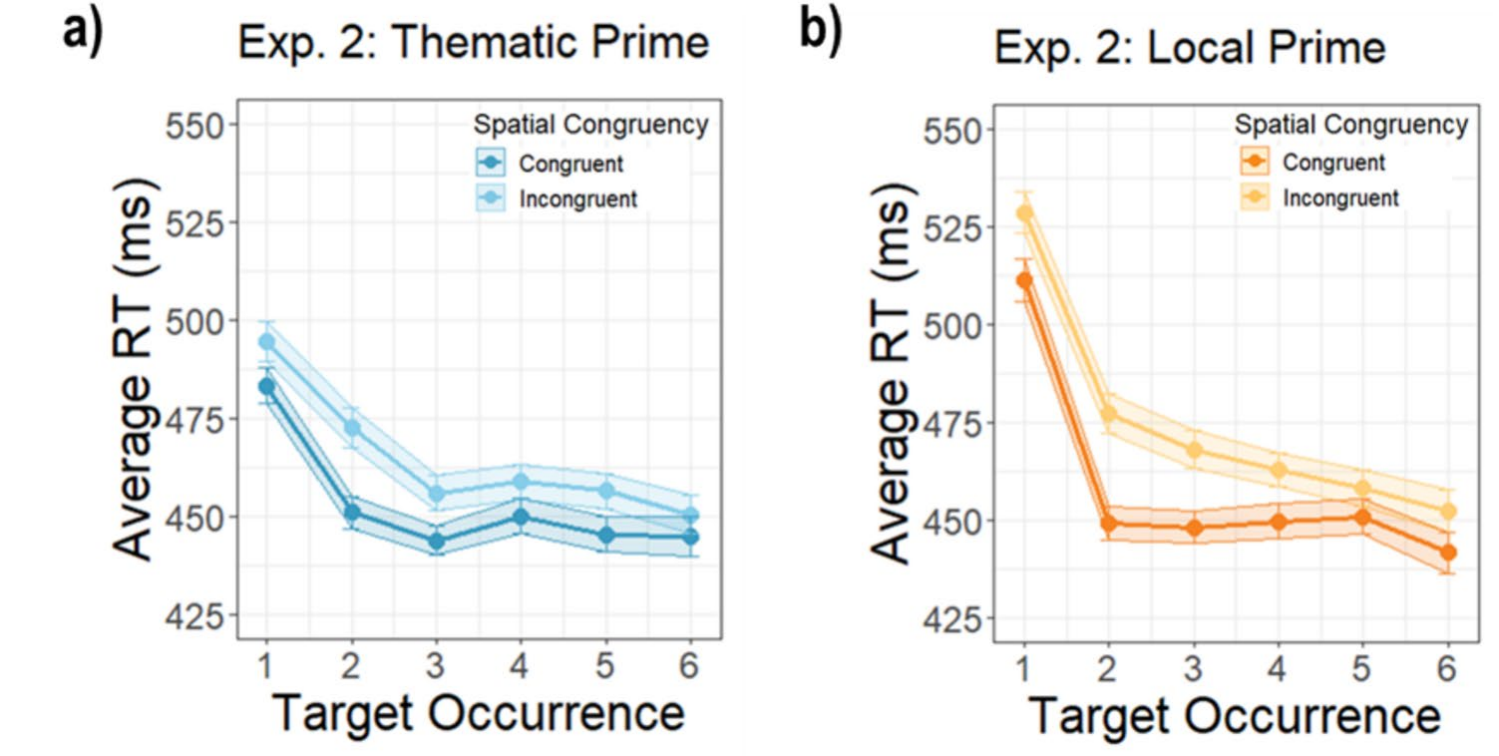
Average reaction times across target occurrences for Experiment 2. Panel **a)** depicts the average RTs for thematic prime trials across target and panel **b)** the local prime trials. The shading around lines indicates standard error of the mean. Data are plotted on the same scale as Experiment 1 to facilitate comparison. (Color figure online)

Next, as in Experiment 1, we examined the effect of learning of prime–target pairs over time (Fig. 8a–b). There was a significant interaction between target occurrence and prime condition (β = 5.76, *SE* = 1.56), *t*(12034.443) = 3.69, *p* <.001, showing that there was steeper learning of pairs in the *local* condition. The interaction between target occurrence and spatial congruency, however, was not significant (β = 2.63, *SE* = 1.59), *t*(12049.302) = 1.66, *p* =.097, nor was the three-way interaction between target occurrence, spatial condition, and prime condition (β = −1.53, *SE* = 2.24), *t*(11896.510) = −0.68, *p* =.494. There was also no significant interaction between spatial congruency and prime condition, consistent with the main RT analysis (β = 10.14, *SE* = 8.17), *t*(12042.584) = 1.24, *p* =.215. These results suggest that the congruency effect was similar for both the thematic and local pairs over time in this experiment.

#### Memory for targets in congruent thematic pairs is still better than for those in local pairs

**Fig. 9:**
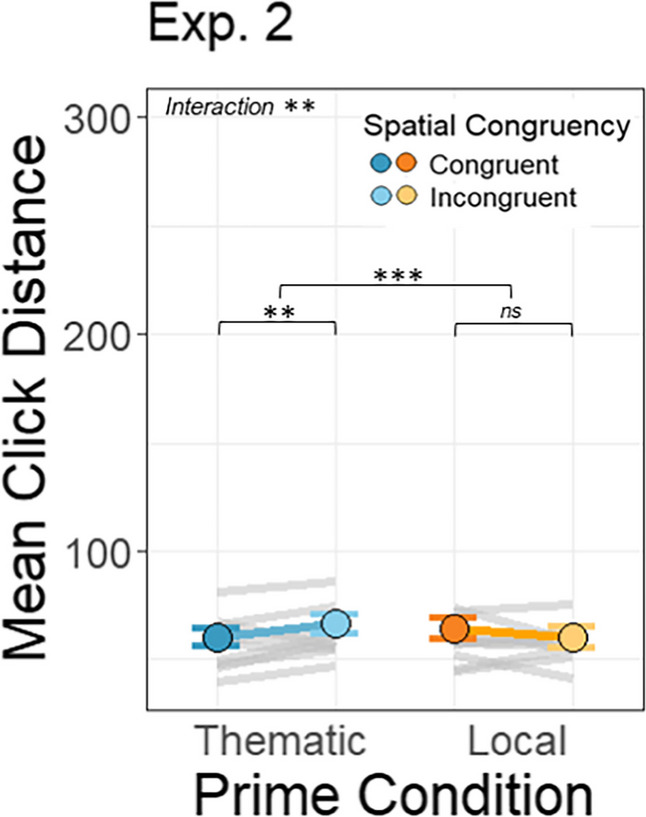
The dots represent the overall mean distance between click responses and the targets’ true location on the screen and the gray lines represent average distances for each target–anchor pairing. Data are plotted on the same scale as for Experiment 1 in order to facilitate comparison. The error bars represent the standard error of the mean across participants for each condition. Data are plotted on the same scale as Experiment 1 to facilitate comparison

There was a significant interaction between prime condition and spatial congruency (β = −6.50, *SE* = 2.05), *t*(4443.24) = −3.17, *p* =.002) (*thematic congruent: M* = 60.17, *SD* = 54.97, *thematic incongruent: M* = 66.53, *SD* = 57.45, *local congruent*: *M* = 64.26, *SD* = 60.43, *local incongruent: M* = 60.47, *SD* = 60.85; Fig. 9). This interaction had the same pattern as that in Experiment 1a and shows an effect of spatial congruency on probe recall in the *thematic* condition, which was reversed in the *local* condition. This suggests that the thematic congruency benefit on target location memory is insensitive to the sequential ordering of the prime and target. Target recall accuracy is higher when the two objects are thematically related and in a congruent configuration, perhaps because the objects form a naturalistic group that bolsters memory for the target location.

There was also significant main effect of prime condition on the click distance from the original target location (β = 7.27, *SE* = 1.57),* t*(4135.09) = 4.63, *p* <.001. However, unlike Experiment 1 the *local* primes yielded slightly smaller click distances than *thematic* primes but this main effect cannot be interpreted outside of the significant interaction. There was no significant main effect of spatial congruency on click distance (β = 0.81, *SE* = 1.47), *t*(4442.84) = 0.55, *p* =.583.

## Discussion

The ability to interpret naturalistic environments depends on our prior knowledge of scene structure and what objects co-occur and function together. This study provides new evidence that anchor objects provide spatial predictions for semantically associated objects. This is important because it reveals an asymmetric role of learned interobject relationships in guiding spatial attention. In Experiments 1a and 1b, we found that targets were found more rapidly when they appeared in their typical location compared with an unusual location relative to a preceding thematically related anchor object; this difference was bigger than for arbitrarily paired prime–target objects with similar physical properties. However, when the roles of the anchor primes and associated targets were reversed in Experiment 2, the difference in the spatial congruency effect between the thematic and local prime conditions disappeared. This indicates that thematic anchor objects are unique in that they act as more consistent spatial predictors for the relative location of associated objects as well as their identity (Võ et al., 2019) and that this spatial prediction is asymmetrical. The unique ability of anchor objects to predict the location of semantically associated objects goes beyond both natural statistics (e.g., “small things go on top of big things”), and semantic relatedness (e.g., pots and stoves are often found together).

It is well established that related objects prime each other’s identity. For example, Collins and Quillian (1969) proposed a model of semantic memory in which related objects are conceptualized as nodes within a connected network. Conceptual priming occurs when one node is activated and spreads activation to connected nodes (Collins & Loftus, 1975; McClelland & Rogers, 2003). Although most of the work on semantic knowledge has focused on the organization of taxonomic relationships, defined by natural categories (e.g., fruits, apples, oranges), semantic relationships can also be thematic in nature and defined by co-occurrence in events or natural situations (e.g., dogs and leash; Estes et al., 2011; Green & Hummel, 2006; Mirman et al., 2017). Thematic associations are particularly interesting in the visual domain because they are evident in real-world scene statistics and drive the distribution of attention (Collegio et al., 2019; Gayet & Peelen, 2022; Koehler & Eckstein, 2017; Lerebourg et al., 2024; Nah & Geng, 2022; Peelen et al., 2023). Our results are generally consistent with these findings, but also provide new direct evidence that certain objects serve as spatial cues for the location of smaller semantically related objects.

In visual scenes, anchor objects are large and have highly reliable positions. They may serve as particularly strong spatial cues because they form a “scene phrase” within which smaller objects are participating elements (e.g., bathroom sink is an anchor for toothbrushes, toothpaste, soap; Turini & Võ, 2022). Anchor objects have been shown to speed search for targets by creating scene structure that guides eye, head, and body movements (Boettcher et al., 2018; Helbing et al., 2022). In contrast, while smaller objects still prime the identity of semantic associates, they may be less effective in producing spatial predictions because their own locations vary more within scenes and are therefore provide less reliable information about the relative location of other objects. Our data confirm this by showing that targets preceded by a thematic anchor were identified faster when they appeared in the normative location (pot on top of a stove) than when they violated expectations (pot on in front of the stove) suggesting that the spatial configuration was encoded directly in the object-to-object representations even outside of a scene.

The advantage afforded by the spatial prediction in searching for the target, given by evidence of differing results in Experiments 1 and 2, can be contrasted with the results from the memory probes. The pattern of memory probes from Experiment 1a and 2 were similar and showed more accurate memory localization of the target when it appeared in a congruent configuration with its thematic prime compared to the local condition prime–target pairs. This suggests that the precision of memory for the target location was boosted when it was preceded by a thematically related prime, perhaps because the natural configuration allowed the two objects to form a perceptual or mnemonic group (Clement et al., 2019; Green & Hummel, 2006; Gronau et al., 2008; Mack & Eckstein, 2011; Nah & Geng, 2022; Nah et al., 2021; Shomstein et al., 2019).

Together, these results demonstrate that anchor objects have a special role in enhancing search for small targets because they generate spatial predictions that optimize attentional guidance and visual search efficiency. Anchor objects appear to be unique in creating expectations of “what” nearby objects are, and “where” they are located. Future research could explore if anchors are truly unique or if object pairs with various levels of semantic relatedness—ranging from weak to strong—generate spatial predictions of varying strengths. Overall, these findings contribute to a broader understanding of how semantic knowledge is used to efficiently navigate and locate target objects in complex and cluttered environments.

## Supplementary Information

Below is the link to the electronic supplementary material.

Supplementary file1 (DOCX 411 KB)

## Data Availability

The materials, primary data, and scripts used in these experiments are accessible on the Open Science Framework (OSF) at: https://osf.io/m34bf/

## Code availability

Data analyses were conducted using custom scripts in RStudio 4.3.1, and these scripts, which allow full reproduction of the presented analyses and Figures, are also available on OSF (https://osf.io/m34bf/).

## Declarations

### Open practices statement

All study materials and primary data collected for Experiments 1ab and 2 are publicly available on OSF (https://osf.io/m34bf/). The analysis code used for all experiments is also available on OSF (https://osf.io/m34bf/).

## References

[CR1] Bates, D., Mächler, M., Bolker, B., & Walker, S. (2015). Fitting linear mixed-effects models using lme4. *Journal of Statistical Software,**67*, 1–48. 10.18637/jss.v067.i01

[CR2] Biederman, I. (1972). Perceiving real-world scenes. *Science,**177*(4043), 77–80. 10.1126/science.177.4043.77 5041781 10.1126/science.177.4043.77

[CR3] Boettcher, S. E. P., Draschkow, D., Dienhart, E., & Võ, M.L.-H. (2018). Anchoring visual search in scenes: Assessing the role of anchor objects on eye movements during visual search. *Journal of Vision,**18*(13), Article 11. 10.1167/18.13.11 30561493 10.1167/18.13.11

[CR4] Brockmole, J. R., Castelhano, M. S., & Henderson, J. M. (2006). Contextual cueing in naturalistic scenes: Global and local contexts. *Journal of Experimental Psychology: Learning, Memory, and Cognition,**32*(4), 699–706. 10.1037/0278-7393.32.4.699 16822141 10.1037/0278-7393.32.4.699

[CR5] Castelhano, M. S., & Krzyś, K. (2020). Rethinking space: A review of perception, attention, and memory in scene processing. *Annual Review of Vision Science,**6*, 563–586. 10.1146/annurev-vision-121219-081745 32491961 10.1146/annurev-vision-121219-081745

[CR6] Clement, A., O’Donnell, R. E., & Brockmole, J. R. (2019). The functional arrangement of objects biases gaze direction.* Psychonomic Bulletin & Review, 26*(4), 1266–1272. 10.3758/s13423-019-01607-8 10.3758/s13423-019-01607-8 31044360

[CR7] Collegio, A. J., Nah, J. C., Scotti, P. S., & Shomstein, S. (2019). Attention scales according to inferred real-world object size. *Nature Human Behaviour,**3*(1), 40–47. 10.1038/s41562-018-0485-2 30932061 10.1038/s41562-018-0485-2

[CR8] Collins, A. M., & Loftus, E. F. (1975). A spreading-activation theory of semantic processing. *Psychological Review,**82*(6), 407–428. 10.1037/0033-295X.82.6.407

[CR9] Collins, A. M., & Quillian, M. R. (1969). Retrieval time from semantic memory. *Journal of Verbal Learning and Verbal Behavior,**8*(2), 240–247. 10.1016/S0022-5371(69)80069-1

[CR10] Draschkow, D., & Võ, M.L.-H. (2017). Scene grammar shapes the way we interact with objects, strengthens memories, and speeds search. *Scientific Reports,**7*, Article 16471. 10.1038/s41598-017-16739-x 29184115 10.1038/s41598-017-16739-x PMC5705766

[CR11] Estes, Z., Golonka, S., & Jones, L. L. (2011). Thematic thinking: The apprehension and consequences of thematic relations. In B. H. Ross (Ed.), *Psychology of learning and motivation* (Vol. 54, pp. 249–294). Academic Press. 10.1016/B978-0-12-385527-5.00008-5

[CR12] Gayet, S., & Peelen, M. V. (2022). Preparatory attention incorporates contextual expectations. *Current Biology,**32*(3), 687-692.e6. 10.1016/j.cub.2021.11.062 34919809 10.1016/j.cub.2021.11.062

[CR13] Green, C., & Hummel, J. E. (2006). Familiar interacting object pairs are perceptually grouped. *Journal of Experimental Psychology. Human Perception and Performance,**32*(5), 1107–1119. 10.1037/0096-1523.32.5.1107 17002525 10.1037/0096-1523.32.5.1107

[CR14] Gronau, N., Neta, M., & Bar, M. (2008). Integrated contextual representation for objects’ identities and their locations. *Journal of Cognitive Neuroscience,**20*(3), 371–388. 10.1162/jocn.2008.20027 18004950 10.1162/jocn.2008.20027

[CR15] Hayes, T. R., & Henderson, J. M. (2019). Scene semantics involuntarily guide attention during visual search. *Psychonomic Bulletin & Review,**26*(5), 1683–1689. 10.3758/s13423-019-01642-5 31342407 10.3758/s13423-019-01642-5

[CR16] Helbing, J., Draschkow, D., & Võ, M.L.-H. (2022). Auxiliary scene-context information provided by anchor objects guides attention and locomotion in natural search behavior. *Psychological Science,**33*(9), 1463–1476. 10.1177/09567976221091838 35942922 10.1177/09567976221091838

[CR17] Henderson, J. M., Weeks, P. A., Jr., & Hollingworth, A. (1999). The effects of semantic consistency on eye movements during complex scene viewing. *Journal of Experimental Psychology. Human Perception and Performance,**25*(1), 210–228. 10.1037/0096-1523.25.1.210

[CR18] Koehler, K., & Eckstein, M. P. (2017). Beyond scene gist: Objects guide search more than scene background. *Journal of Experimental Psychology. Human Perception and Performance,**43*(6), 1177–1193. 10.1037/xhp0000363 28287759 10.1037/xhp0000363

[CR19] Krugliak, A., Draschkow, D., Võ, M.L.-H., & Clarke, A. (2024). Semantic object processing is modulated by prior scene context. *Language, Cognition and Neuroscience,**39*(8), 962–971. 10.1080/23273798.2023.2279083 39404678 10.1080/23273798.2023.2279083 PMC7616603

[CR20] Kumle, L., Võ, M. L.-H., & Draschkow, D. (2021). Estimating power in (generalized) linear mixed models: An open introduction and tutorial in R. *Behavior Research Methods, 53*(6), 2528–2543. 10.3758/s13428-021-01546-0 10.3758/s13428-021-01546-0 PMC8613146 33954914

[CR21] Lerebourg, M., de Lange, F. P., & Peelen, M. V. (2024). Attentional guidance through object associations in visual cortex. *Science Advances,**10*(41), Article eado6226. 10.1126/sciadv.ado6226 41544144 10.1126/sciadv.ado6226 PMC11468920

[CR22] Mack, S. C., & Eckstein, M. P. (2011). Object co-occurrence serves as a contextual cue to guide and facilitate visual search in a natural viewing environment. *Journal of Vision,**11*(9), 9. 10.1167/11.9.9 10.1167/11.9.9 21856869

[CR23] McClelland, J. L., & Rogers, T. T. (2003). The parallel distributed processing approach to semantic cognition. *Nature Reviews Neuroscience,**4*(4), 310–322. 10.1038/nrn1076 12671647 10.1038/nrn1076

[CR24] Meteyard, L., & Davies, R. A. I. (2020). Best practice guidance for linear mixed-effects models in psychological science. *Journal of Memory and Language,**112*, Article 104092. 10.1016/j.jml.2020.104092

[CR25] Mirman, D., Landrigan, J.-F., & Britt, A. E. (2017). Taxonomic and thematic semantic systems. *Psychological Bulletin,**143*(5), 499–520. 10.1037/bul0000092 28333494 10.1037/bul0000092 PMC5393928

[CR26] Nah, J. C., & Geng, J. J. (2022). Thematic object pairs produce stronger and faster grouping than taxonomic pairs. *Journal of Experimental Psychology. Human Perception and Performance,**48*, 1325–1335. 10.1037/xhp0001031 36442038 10.1037/xhp0001031

[CR27] Nah, J. C., Malcolm, G. L., & Shomstein, S. (2021). Task-Irrelevant Semantic Properties of Objects Impinge on Sensory Representations within the Early Visual Cortex. *Cerebral Cortex Communications, 2*(3), tgab049. 10.1093/texcom/tgab049 10.1093/texcom/tgab049 PMC8382923 34447936

[CR28] Peacock, C. E., Hayes, T. R., & Henderson, J. M. (2019). Meaning guides attention during scene viewing, even when it is irrelevant. *Attention, Perception & Psychophysics,**81*(1), 20–34. 10.3758/s13414-018-1607-7 10.3758/s13414-018-1607-7 PMC6384129 30353498

[CR29] Peelen, M. V., Berlot, E., & de Lange, F. P. (2023). Predictive processing of scenes and objects. *Nature Reviews Psychology,**3*, 13. 10.1038/s44159-023-00254-0 38989004 10.1038/s44159-023-00254-0 PMC7616164

[CR30] Shomstein, S., Malcolm, G. L., & Nah, J. C. (2019). Intrusive effects of task-irrelevant information on visual selective attention: Semantics and size. *Current Opinion in Psychology,**29*, 153–159. 10.1016/j.copsyc.2019.02.008 30925285 10.1016/j.copsyc.2019.02.008

[CR31] Torralba, A., Oliva, A., Castelhano, M. S., & Henderson, J. M. (2006). Contextual guidance of eye movements and attention in real-world scenes: The role of global features in object search. *Psychological Review,**113*(4), 766–786. 10.1037/0033-295X.113.4.766 17014302 10.1037/0033-295X.113.4.766

[CR32] Turini, J., & Võ, M.L.-H. (2022). Hierarchical organization of objects in scenes is reflected in mental representations of objects. *Scientific Reports,**12*(1), Article 20068. 10.1038/s41598-022-24505-x 36418411 10.1038/s41598-022-24505-x PMC9684142

[CR33] Võ, M.L.-H., & Wolfe, J. M. (2013). Differential ERP signatures elicited by semantic and syntactic processing in scenes. *Psychological Science,**24*(9), 1816–1823. 10.1177/0956797613476955 23842954 10.1177/0956797613476955 PMC4838599

[CR34] Võ, M.L.-H., Boettcher, S. E., & Draschkow, D. (2019). Reading scenes: How scene grammar guides attention and aids perception in real-world environments. *Current Opinion in Psychology,**29*, 205–210. 31051430 10.1016/j.copsyc.2019.03.009

