# Supplementary material for: Anchor objects guide spatial attention during visual search
Source: Atten Percept Psychophys. 2025 Dec 2;88(1):19. doi: 10.3758/s13414-025-03198-0 (PMC12672618; doi:10.3758/s13414-025-03198-0)
Supplement: Supplementary file 1 — Supplementary file1 (DOCX 411 KB) [file 13414_2025_3198_MOESM1_ESM.docx]

# **Supplemental**

**Table 1**

*Experiment 1a: Participant Demographics*

| Demographic  Category | Count | |
| --- | --- | --- |
|  | *n* |  |
| Gender |  |  |
| Female | 165 |  |
| Male | 40 |  |
| Non-Binary | 5 |  |
| Race |  |  |
| American Indian/Alaskan Native | 1 |  |
| African American or Black | 3 |  |
| Asian | 110 |  |
| White | 53 |  |
| More than One Race | 15 |  |
| Not Listed | 28 |  |
| Other | 1 |  |
| Ethnicity |  |  |
| Hispanic or Latino | 56 |  |
| Not Hispanic or Latino | 156 |  |
|  |  |  |
|  |  |  |

*Fig. N* = 210. Participants were on average 19.7 years old.

**Table 2**

*Experiment 1b: Participant Demographics*

| Demographic  Category | Count | |
| --- | --- | --- |
|  | *n* |  |
| Gender |  |  |
| Female | 118 |  |
| Male | 38 |  |
| Non-Binary | 1 |  |
| Race |  |  |
| American Indian/Alaskan Native | 1 |  |
| African American or Black | 4 |  |
| Asian | 64 |  |
| White | 48 |  |
| More than One Race | 15 |  |
| Not Listed | 23 |  |
| Other | 2 |  |
| Ethnicity |  |  |
| Hispanic or Latino | 42 |  |
| Not Hispanic or Latino | 115 |  |
|  |  |  |
|  |  |  |

*Fig. N* = 157. Participants were on average 19.9 years old

**Table 3**

*Experiment 2: Participant Demographics*

| Demographic  Category | Count | |
| --- | --- | --- |
|  | *n* |  |
| Gender |  |  |
| Female | 122 |  |
| Male | 30 |  |
| Non-Binary | 6 |  |
| Race |  |  |
| American Indian/Alaskan Native | 1 |  |
| African American or Black | 4 |  |
| Asian | 58 |  |
| White | 56 |  |
| More than One Race | 13 |  |
| Not Listed | 24 |  |
| Other | 2 |  |
| Ethnicity |  |  |
| Hispanic or Latino | 41 |  |
| Not Hispanic or Latino | 117 |  |
|  |  |  |
|  |  |  |

*Fig. N* = 158. Participants were on average 19.8 years old

**Table 4**

*Experiment 1a: Reaction Times Pairwise Comparisons*

| Contrast |  | β | SE | Z | *P* |
| --- | --- | --- | --- | --- | --- |
| Local Incongruent - Thematic Incongruent |  | -11.1 | 1.57 | -7.07 | <.0001 |
| Local Incongruent - Local Congruent |  | 6.38 | 1.56 | 4.09 | <.001 |
| Local Incongruent -Thematic Congruent |  | 8.13 | 1.57 | 5.17 | <.0001 |
| Thematic Incongruent - Local Congruent |  | 17.50 | 1.57 | 11.17 | <.0001 |
| Thematic Incongruent - Thematic Congruent |  | 19.24 | 1.58 | 12.19 | <.0001 |
| Local Congruent - Thematic Congruent |  | 1.74 | 1.57 | 1.11 | 0.681 |
|  |  |  |  |  |  |

*Estimated marginal means pairwise comparisons.*

*P value adjustment: tukey method for comparing a family of 4 estimates.*

**Table 5**

*Experiment 1a: Accuracy Pairwise Comparisons*

| Contrast |  | β | SE | Z | *P* |
| --- | --- | --- | --- | --- | --- |
| Local Incongruent - Local Congruent |  | -0.03 | 0.1 | -0.29 | 0.771 |
| Thematic Incongruent - Thematic Congruent |  | -0.21 | 0.95 | -2.18 | 0.029 |
|  |  |  |  |  |  |

*Estimated marginal means pairwise comparisons.*

*P value adjustment: tukey method for comparing a family of 4 estimates.*

**Table 6**

*Experiment 1b: Reaction Times Pairwise Comparisons*

| Contrast |  | β | SE | Z | *P* |
| --- | --- | --- | --- | --- | --- |
| Local Incongruent - Thematic Incongruent |  | 10.92 | 2.46 | -4.43 | <.0001 |
| Local Incongruent - Local Congruent |  | 8.28 | 2.45 | 3.38 | 0.004 |
| Local Incongruent -Thematic Congruent |  | 5.52 | 2.46 | 2.25 | 0.111 |
| Thematic Incongruent - Local Congruent |  | 19.20 | 2.46 | 7.81 | <.0001 |
| Thematic Incongruent - Thematic Congruent |  | 16.44 | 2.46 | 6.68 | <.0001 |
| Local Congruent - Thematic Congruent |  | -2.76 | 2.45 | -1.13 | 0.674 |
|  |  |  |  |  |  |

*Estimated marginal means pairwise comparisons.*

*P value adjustment: tukey method for comparing a family of 4 estimates.*

**Table 7**

*Experiment 1b: Accuracy Pairwise Comparisons*

| Contrast |  | β | SE | Z | *P* |
| --- | --- | --- | --- | --- | --- |
| Local Incongruent - Local Congruent |  | -0.05 | 0.22 | -0.244 | 0.808 |
| Thematic Incongruent - Thematic Congruent |  | -0.05 | 0.17 | -0.29 | 0.775 |
|  |  |  |  |  |  |

*Estimated marginal means pairwise comparisons.*

*P value adjustment: tukey method for comparing a family of 4 estimates.*

**Table 8**

*Experiment 1a: Memory Probe Pairwise Comparisons*

| Contrast |  | β | SE | Z | *P* |
| --- | --- | --- | --- | --- | --- |
| Local Incongruent - Local Congruent |  | -7.22 | 0.85 | -8.53 | <.0001 |
| Thematic Incongruent - Thematic Congruent |  | 33.37 | 0.86 | 38.97 | <.0001 |
|  |  |  |  |  |  |

*Estimated marginal means pairwise comparisons.*

*P value adjustment: tukey method for comparing a family of 4 estimates.*

**Table 9**

*Experiment 1b: Memory Probe Pairwise Comparisons*

| Contrast |  | β | SE | Z | *P* |
| --- | --- | --- | --- | --- | --- |
| Local Incongruent - Local Congruent |  | -5.58 | 1.76 | -3.16 | 0.009 |
| Thematic Incongruent - Thematic Congruent |  | 1.90 | 1.75 | 1.09 | 0.698 |
|  |  |  |  |  |  |

*Estimated marginal means pairwise comparisons.*

*P value adjustment: tukey method for comparing a family of 4 estimates.*

**Table 10**

*Experiment 2: Reaction Times Pairwise Comparisons*

| Contrast |  | β | SE | Z | *P* |
| --- | --- | --- | --- | --- | --- |
| Local Incongruent - Thematic Incongruent |  | 11.62 | 2.59 | 4.49 | <.0001 |
| Local Incongruent - Local Congruent |  | 17.57 | 2.60 | 6.76 | <.0001 |
| Local Incongruent -Thematic Congruent |  | 23.17 | 2.58 | 8.96 | <.0001 |
| Thematic Incongruent - Local Congruent |  | 5.95 | 2.58 | 2.31 | 0.096 |
| Thematic Incongruent - Thematic Congruent |  | 11.54 | 2.56 | 4.51 | <.0001 |
| Local Congruent - Thematic Congruent |  | 5.59 | 2.57 | 2.17 | 0.131 |
|  |  |  |  |  |  |

*Estimated marginal means pairwise comparisons.*

*P value adjustment: Tukey method for comparing a family of 4 estimates.*

**Table 11**

*Experiment 2: Accuracy Pairwise Comparisons*

| Contrast |  | β | SE | Z | *P* |
| --- | --- | --- | --- | --- | --- |
| Local Incongruent - Local Congruent |  | -0.12 | 0.20 | -0.59 | 0.557 |
| Thematic Incongruent - Thematic Congruent |  | -0.00 | 0.21 | -0.02 | 0.983 |
|  |  |  |  |  |  |

*Estimated marginal means pairwise comparisons.*

*P value adjustment: Tukey method for comparing a family of 4 estimates.*

**Table 12**

*Experiment 2: Memory Probe Pairwise Comparisons*

| Contrast |  | β | SE | Z | *P* |
| --- | --- | --- | --- | --- | --- |
| Local Incongruent - Local Congruent |  | -0.81 | 1.47 | -0.55 | 0.947 |
| Thematic Incongruent - Thematic Congruent |  | 5.69 | 1.43 | 3.98 | <.001 |
|  |  |  |  |  |  |

*Estimated marginal means pairwise comparisons.*

*P value adjustment: tukey method for comparing a family of 4 estimates.*

**Preliminary Experiment**

Note: This preliminary experiment was used to determine sample size for Experiment 1a.

**Method**

***Participants***

A total of 130 undergraduate students from the University of California, Davis were recruited through the UC Davis SONA system to participate in this study. In exchange for their participation, students received course credit. Data were collected online using the Testable platform (https://www.testable.org/) until we obtained a sample of 130 participants after exclusion criteria were applied. 16 participants were excluded from the analysis because of poor performance in standard search trials (accuracy was below 80%). Participants were randomly assigned to one of three experimental groups, each group experiencing two out of the three possible experimental conditions. The distribution of participants was as follows: *Thematic & Local* (n = 44), *Thematic & Random* (n = 43), and *Local & Random* (n = 43). A total of 92 participants identified as female, 32 identified as male, 6 identified as non-binary, and the average age was 20.5 years. All participants had normal or corrected-to-normal vision and were naive to the purpose of the experiment. Each participant provided informed consent in accordance with the guidelines set by the University of California, Davis Institutional Review Board (IRB).

Additionally, a separate group of participants (n = 146) engaged in a judgment task where they rated the strength of thematic co-occurrence between pairs of objects used in the main experiment. This task was also conducted using the Testable platform, and participants were recruited through the SONA system and received course credit for their participation. Participants saw all object pairings in a randomized order for all conditions. The same requirements for participation in the main experiment applied for the judgment task.

***Stimuli and Apparatus***

Our stimulus set consisted of a subset of images used in Nah & Geng (2023). They tested each image for luminance, color, and curvilinear and rectilinear values to rule out systematic differences between semantic conditions of interest (Malcolm et al., 2016). Our stimuli for experiment 1 were images of real world objects. All stimuli were presented on a white background.

In the separate judgment task, participants evaluated each object pairing across conditions and rated on a 6-point scale how likely the objects were to appear together in real life, with 1 indicating ‘extremely unlikely’ (i.e., no thematic relationship) and 6 indicating ‘extremely likely’ (i.e., strong thematic relationship). These ratings were used to assess the thematic strength of each object pairing. For the main experiment, pairs selected for the thematic condition had greater ratings (M = 5.78, SD = 0.17, SE = 0.032) than the local (M = 1.94, SD = 0.18, SE = 0.056) than the random condition (M = 2.30, SD = 0.32, SE = 0.034). This confirmed that object pairs were classified correctly. In total, ten thematic, ten local, and ten random object pairings were utilized in the experiment.

***Design and Procedure***

We manipulated the semantic relatedness of target object pairs in a Rapid Serial Visual Presentation (RSVP) task. At the start of each trial, the target word (e.g., "Pot") was presented for 2000 ms. Following this, each trial featured 2 to 5 displays, each containing two lateralized objects on either side of a central fixation cross. Each object display appeared for 350 ms, with a 900 ms interval between displays. During the series of displays, an object prime would randomly appear at any point (between displays 1-4). The target object would then appear in the following display (e.g., if the prime appeared in display 3, the target would appear in display 4) (see **Fig. 1.**). Notably, the prime would always appear on the same side of the screen that the upcoming target would appear. However, to prevent predictability and maintain attention, stimuli were randomly jittered around the fixation point within an invisible circular pattern.

The prime could be thematically related to the target object, locally related, or completely random. The thematically related prime was an object that would statistically co-occur with the target object (e.g., “stove”). The local prime was an unrelated object that consistently appeared before the target in the task (e.g., "sink"), providing a constant but semantically unrelated cue. In the random condition, different unrelated objects were used as primes for each trial. Participants were divided into three groups, each experiencing two conditions: Thematic and Local, Thematic and Random, or Local and Random.

The trials were randomized, meaning participants could encounter either of the two conditions they were assigned on any given trial. Each target was presented nine times, totaling 180 trials per participant. Each object was randomly overlaid with a horizontal (180°) or vertical Gabor patch (90°). Upon locating the target, participants reported the orientation of the Gabor patch by clicking a button on their keyboard (“J” for horizontal and “I” for vertical orientation). Participants completed four practice trials before starting the main task and received accuracy feedback throughout the task. Upon completion, participants filled out a short survey.


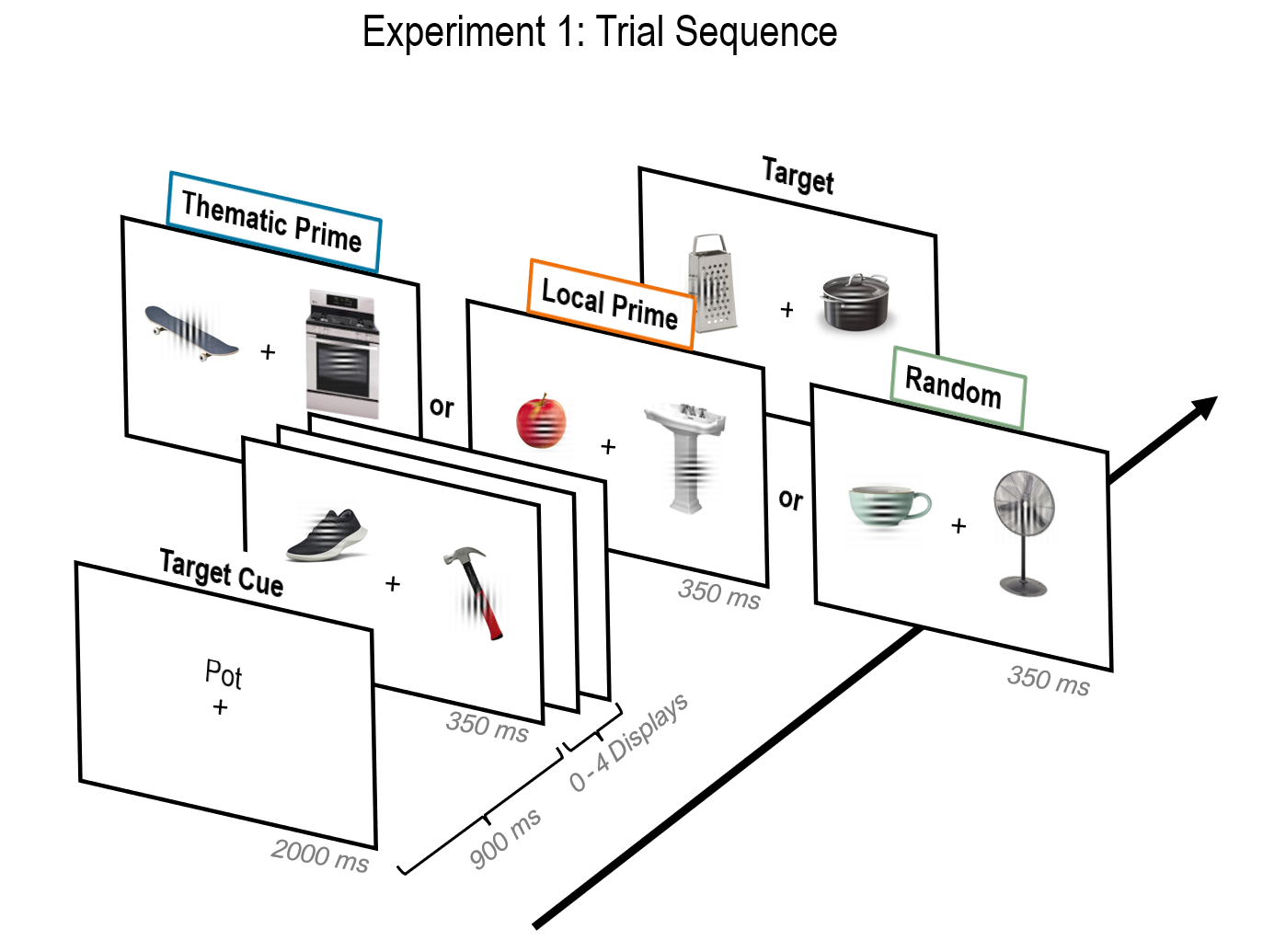


**Fig.1** Trial Sequence. First a target cue (e.g., a pot) appeared, followed by 0–4 “filler” displays. Next, a prime object display appeared with a thematic prime (blue), local prime (orange), or random prime (green). This was followed immediately by a target display with the target appearing on the same side of the fixation as the prime. All displays were visible for 350 ms with a 900 ms ISI. Participants responded by pressing a button to indicate the gabor’s orientation. Note: Object stimuli were jittered in a circular pattern to reduce predictability, and object size is enlarged in this figure for visualization purposes.

***Data Analysis***

Data were extracted from Testable and analyzed using a custom script in R Studio. Reaction time (RT) data were analyzed using a linear mixed-effects regression model with the lme4 package (Bates et al., 2015), specifically using the lmer function. For our mixed-effects regression, the outcome variable was RT, the fixed effects predictor was the prime condition (thematic, local, and random). Dummy codes were used to represent the prime conditions, with random being coded as 0 and local coded as 1. The thematic condition served as the baseline and was compared against the other conditions. Random effects were also included for participant groups and individual participants. These were added to control for subject group and individual participant variability. Only incorrect trials, and trials with RTs less than 200 ms (anticipatory responses) were removed from the RT analysis.

Accuracy data were analyzed using a logistic mixed-effects logistic regression model with the lme4 package and glmer function, where accuracy was the outcome variable and the model included the same fixed and random effects as the RT analysis. Additionally, model comparisons and inference for fixed effects were conducted using the lmerTest package (Kuznetsova et al., 2017)

**Results**

***Reaction Time Analysis***

The linear mixed-effects regression of the prime condition revealed a significant main effect on reaction time (RT). Participants responded significantly faster in the *thematic* prime condition compared to the *local* condition (β = -19.67, SE = 2.53, t(15955.47) = -7.77, p < .001) and the *random* condition (β = -18.02, SE = 2.61, t(15925.65) = -6.91, p < .001). The difference in RT between the *local* and *random* conditions was not statistically significant (β = -1.65, SE = 2.56, t(15960.03) = -0.65, p = .518). Participants identified targets fastest when primed with a thematically related object, compared to when they were primed with a random or local object (**Fig. 2a**). This suggests that holding the target in mind led the thematically related object to capture attention, which then primed the detection of the cued target.

***Accuracy Analysis***

Participants in the *thematic* condition demonstrated significantly higher accuracy compared to the *random* condition (β = 0.31, SE = 0.04, z = 6.86, p < .001), indicating that thematically related primes led to more accurate target identification. Additionally, there was no significant difference in accuracy between the *local* condition and the *random* condition (β = 0.05, SE = 0.04, z = 1.31, p = .191), suggesting that locally associated primes did not significantly impact accuracy relative to random primes (**Fig. 2b**). These results complement those from RT and indicate that a thematic prime improved target detection and identification.

**Fig.2** Average Reaction Times and Accuracy Across Conditions. Colored bars represent mean values for each condition. The error bars represent the standard error of the mean across participants in each condition.


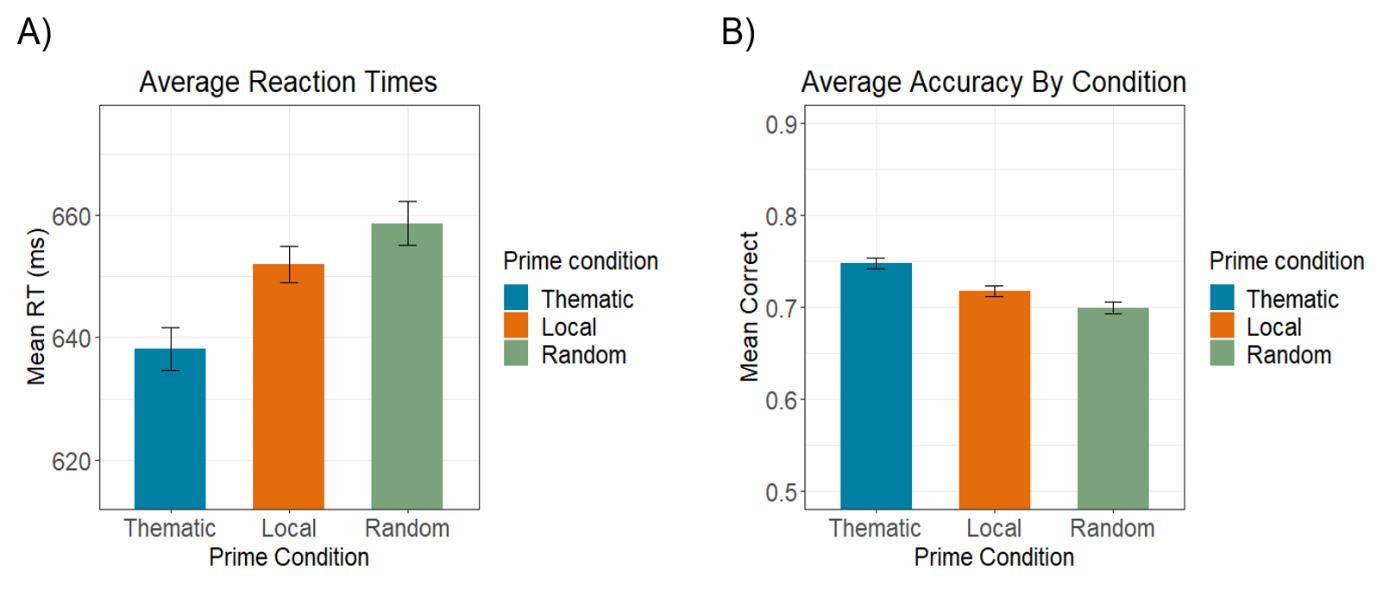


**Discussion**

The findings suggest that semantically related objects capture attention and "prime" the upcoming target. Participants responded faster and more accurately when targets were preceded by a thematically related prime compared to local or random prime. This confirms our expectation based on previous research that semantic relatedness should enhance both the speed and accuracy of target identification. However, this facilitation could be due to two non-mutually exclusive mechanisms: the reciprocal semantic priming between the two objects, and/or the prime generating a spatial prediction for where the target might appear. In this experiment, we could not distinguish between these two possibilities because the target consistently appeared on the same side of the screen as the prime. Thus, it is unclear whether the prime acted as a spatial predictor, over and above acting as a semantic prime.
